# Tobacco-Specific Nitrosamines in Electronic Cigarettes: Comparison between Liquid and Aerosol Levels

**DOI:** 10.3390/ijerph120809046

**Published:** 2015-07-31

**Authors:** Konstantinos E. Farsalinos, Gene Gillman, Konstantinos Poulas, Vassilis Voudris

**Affiliations:** 1 Department of Cardiology, Onassis Cardiac Surgery Center, Sygrou 356, Kallithea 17674, Greece; E-Mail: vvoudris@otenet.gr; 2 Department of Pharmacy, University of Patras, Rio 26500, Greece; E-Mail: kpoulas@otenet.gr; 3 Enthalpy Analytical, Inc., 800 Capitola Drive, Suite 1, NC 27713, USA; E-Mail: gene.gillman@enthalpy.com

**Keywords:** electronic cigarettes, tobacco-specific nitrosamines, aerosol, nicotine, tobacco, smoking

## Abstract

*Introduction*: Although electronic cigarette (EC) liquids contain low levels of tobacco-specific nitrosamines (TSNAs), studies evaluating the levels emitted to the aerosol are scarce. The purpose of this study was to compare the levels of TSNAs between liquids and generated aerosol. *Methods*: Three EC liquids were obtained from the market. An additional (spiked) sample was prepared by adding known amounts of standard TSNAs solutions to one of the obtained liquids. N-nitrosonornicotine (NNN), N-nitrosoanatabine (NAT), N-nitrosoanabasine (NAB) and 4-(methylnitrosamino)1-(3-pyridyl)-1-butanone (NNK) were measured. Three 100-puff sets from each liquid were trapped in filter pads and were subsequently analyzed for the presence of TSNAs. The expected levels of TSNAs (calculated based on the liquid consumption) were compared with the measured levels in the aerosol. *Results*: Only NAB was found at trace levels in two commercial liquids (1.2 and 2.3 ng/g), while the third contained 1.5 ng/g NAB and 7.7 ng/g NNN. The 100-puff sets resulted in 336–515 mg liquid consumption, with no TSNAs being detected in the aerosol. The spiked sample contained 42.0–53.9 ng/g of each of the TSNAs. All TSNAs were detected in the aerosol with the measured levels being statistically similar to the expected amounts. A significant correlation between expected and measured levels of TSNAs in the aerosol was found (r = 0.83, *p* < 0.001). *Conclusion*: The findings of this study show that exposure of EC users to TSNAs can be accurately assessed based on the levels present in the liquid, without the need to analyze the aerosol.

## 1. Introduction

Electronic cigarettes (ECs) are considered as less harmful alternatives to smoking [1,2]. Most studies have focused on examining EC liquid composition, showing that the levels of toxic chemicals present in EC liquids are by far lower than in tobacco cigarette [1]. Tobacco-specific nitrosamines (TSNAs), which are very potent carcinogenic chemicals [3,4], are present in minute amounts in EC liquids, usually at levels comparable to pharmaceutical nicotine products [5,6,7]. TSNAs are derived from the tobacco leaves. They are naturally occurring compounds found in cured tobacco. They are not present in the green tobacco leaves but are formed during the curing process by nitrosation of amines. There is some controversy as to whether combustion leads to substantial TSNAs formation [8,9,10], however, it seems likely that most TSNAs found in mainstream smoke come from the compounds present in cured tobacco leaves while only a small fraction is derived from pyrolytic synthesis [11]. The levels emitted to tobacco cigarette smoke directly correlate with the levels present in the tobacco leaves, but the absolute levels are usually considerably lower [11].

Studies evaluating TSNAs levels in EC aerosol are scarce. Despite finding low levels in the aerosol [12,13], there is little information as to whether the evaporation process results in additional TSNAs production. Concerns have been expressed in the literature that the heat of evaporation may result in higher levels of TSNAs emitted to the aerosol compared to those present in the liquid [14]. The true exposure of consumers (vapers) to TSNAs (through inhalation of the aerosol) relative to the levels present in the liquids has not been adequately assessed. The purpose of this study was to examine whether TSNAs levels in the EC aerosol exceed the levels present in the liquids.

## 2. Methods

### 2.1. Sample Selection

Three commercially-available EC liquids of tobacco flavor from two of the largest Greek EC companies were bought from vapeshops (Table 1). These liquids were available at different nicotine concentration; we obtained the highest available nicotine concentration (18 mg/mL) since TSNAs in EC liquids are probably contaminants in nicotine and the highest nicotine content is expected to contain the highest possible TSNAs levels. Additionally, an EC device set was bought (Epsilon 1100, Nobacco, Athens, Greece), composed of a 2nd generation (eGo-style) lithium battery with a capacity of 1100 mAh and a tank-type atomizer. The liquid samples were stored in regular room conditions before being sent to the laboratory for analysis.

### 2.2. Protocol Design and Methods of Analysis

The liquid samples were analyzed for N-nitrosonornicotine (NNN), N-nitrosoanatabine (NAT), N-nitrosoanabasine (NAB), and 4-(methylnitrosamino)1-(3-pyridyl)-1-butanone (NNK). A measured amount of isotopically labeled internal standards and an aliquot of EC liquid were added to a measured amount of water and mixed thoroughly. The samples were then subjected to ultra-performance liquid chromatography and quantified via tandem mass spectrometry (UPLC-MS/MS) according to the Cooperation Centre for Scientific Research Relative to Tobacco (CORESTA) method 75. The limit of detection (LOD) of the liquid analysis 7.7 ng/g for NNN, 4.6 ng/g for NAT, 1.5 ng/g for NAB, and 3.7 ng/g for NNK.

Subsequently, 1.5 mL of liquid was introduced into the atomizer and attached to the EC battery device. Aerosol was produced through a smoking machine, taking puffs of 4 s duration and 55 mL volume with an interpuff interval of 30 s. The battery part of the device was fully charged before use, and a new wick-coil replacement head was used for each sample. Three sets of 100 puffs were collected from each EC liquid. The atomizer was weighed before and after each puffs set in order to evaluate liquid consumption. The aerosol from each set was trapped separately in glass fiber filter pads. After the addition of an internal standard, the total particulate matter collected on the glass fiber filter pad was extracted into a measured amount of water. The samples were then subjected to UPLC-MS/MS for analysis of TSNAs, similarly to the method use for liquid analysis. The limit of detection (LOD) of the aerosol analysis was 10 ng for NNN, NAT, NAB, and NNK.

Since EC liquids were expected to contain only minimal amounts of TSNAs, a separate liquid sample (spiked sample) was prepared by adding a known amount of TSNAs standard solutions to one of the commercial liquids (Number 3, Nobacco, Athens, Greece). The final levels of TSNAs were measured in the spiked sample. Subsequently, aerosol was produced (three sets of 100 puffs each), trapped and analyzed similarly to the method presented above.

### 2.3. Statistical Analysis

The expected aerosol TSNAs levels were calculated from the respective liquid levels and the amount of liquid consumed in each puffing set using the formula: *expected TSNAs (ng) = liquid TSNAs (ng/g) x liquid consumption (g)*. Comparison between the expected and measured TSNAs levels in the aerosol was performed by using paired samples t-test, while Pearson’s correlation coefficient was used to examine their association. A two-tailed P value of 0.05 was considered statistically significant, and all analyses were performed using commercially available software (SPSS v.22, Chicago, IL, USA).

## 3. Results

The levels of TSNAs in the commercial and the spiked EC liquids are presented in Table 1. Similarly to previous studies [5,6,7], minimal levels of nitrosamines were found in the liquid samples. Only NAB was found at levels above the LOD in two of the liquids, while one liquid contained small amounts of both NAB and NNN. The spiked sample contained measurable amounts of all four TSNAs.

**Table 1:** Levels of tobacco-specific nitrosamines in the electronic cigarette liquids tested in the study.

Liquid Samples	Company	Nicotine Content (mg/mL)	NNN (ng/g)	NAT (ng/g)	NAB (ng/g)	NNK (ng/g)
Mystique Tobacco Echo	Nobacco, Athens, Greece	18	7.7	ND	1.5	ND
Maxx-Blend	Flavourart, Oleggio, Italy	18	ND	ND	2.3	ND
Numbers Three	Nobacco, Athens, Greece	18	ND	ND	1.2	ND
Spiked Sample ^a^		18	46.9	53.9	45.6	42.0

Notes: Abbreviations. NNN, N-nitrosonornicotine; NAT, N-nitrosoanatabine; NAB, N-nitrosoanabasine; NNK, 4-(methylnitrosamino)1-(3-pyridyl)-1-butanone; ND, not detected. ^a^ Spiked sample was “Numbers Three” in which a known amount of standard solutions of tobacco-specific nitrosamines was added.

**Figure 1:**
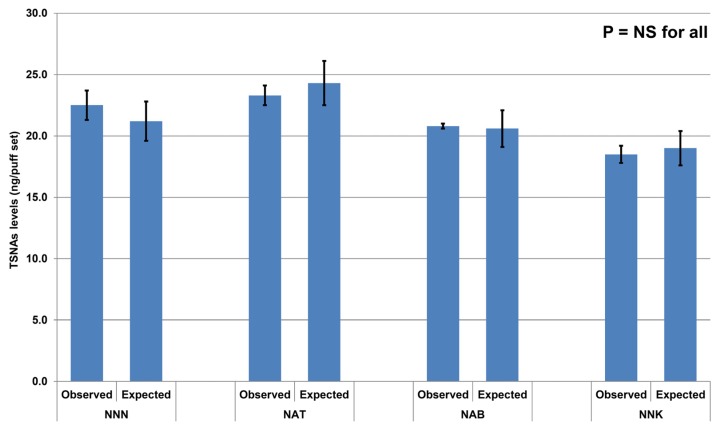
Expected and measured levels of tobacco-specific nitrosamines (TSNAs) in the electronic cigarette aerosol generated from the spiked liquid sample. Bars represent the average from three sets of 100 puffs each while error bars represent standard deviation. For abbreviations, see Table 1.

The 100-puff sets of commercial liquids resulted in the average liquid consumption ranging from 336 to 515 mg for each of the liquid. Due to the very low levels of TSNAs in the commercial liquids, the respective aerosol samples contained, as expected, no TSNAs above the LOD. The analysis of the aerosol generated from the spiked sample is shown in Figure 1.

**Figure 2:**
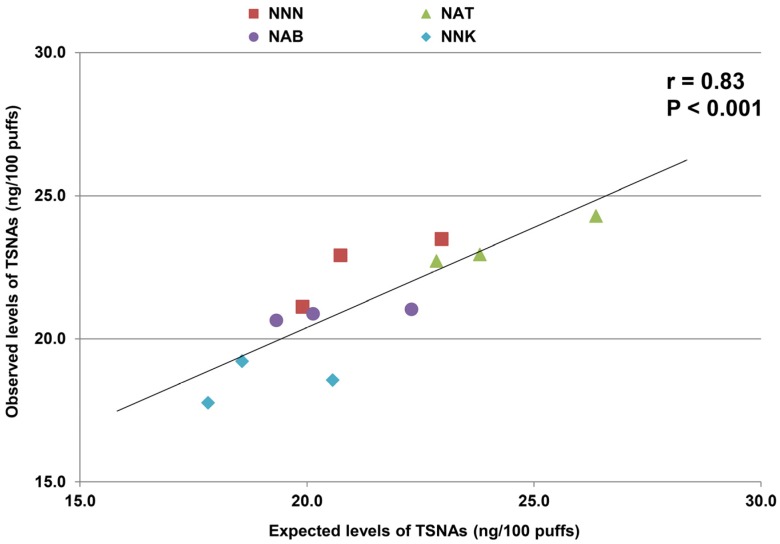
Correlation between observed and expected levels of all tobacco-specific nitrosamines in the aerosol generated from the spiked liquid sample.

The average liquid consumption from the three puffing sets was 452 mg. All TSNAs were detected in the aerosol, at levels that were not statistically different from the expected amounts (*p* = NS). A strong correlation between expected and measured amount of TSNAs was observed (r = 0.83, *p* < 0.001, Figure 2).

## 4. Discussion

This is the first study which directly compared the levels of TSNAs between EC liquids and the respective aerosol. The main finding of the study was that very low levels of TSNAs were present in the EC aerosol, while the results of the spiked sample (prepared with TSNAs added to the original EC liquid) indicated that all TSNAs present in the liquid were readily delivered to the aerosol.

Smoking is a major preventable risk factor for a variety of diseases. Tobacco cigarette smoke contains more than 7000 chemicals many of which are classified as carcinogens. TSNAs have been characterized as potent carcinogens in cigarette smoke. Part of the TSNAs present in mainstream cigarette smoke come from transfer from the filler of a combusting cigarette [15] while another small portion is synthesized during the smoking process [16,17]. The levels of TSNAs in the EC liquids are minimal and by far lower compared to tobacco [5,7,18]. This is probably attributed to the use of pharmaceutical grade nicotine that most manufacturers claim to use. This grade of nicotine is highly purified to remove the majority of impurities, including TSNAs. However, there were no published reports evaluating if additional TSNAs can be formed during EC aerosol production. This is important since ECs are used in aerosol and not in liquid form, and heating is involved in the evaporation process. The study herein verifies that the levels present in the aerosol are similar to those present in the liquid. Therefore, the analysis of TSNAs levels in the liquid would be enough to estimate the exposure of consumers to these substances, without the need to perform more complex and expensive analyses in the aerosol.

A limitation of our study is that, although we found equal amounts of TSNAs in liquid and aerosol, it was not designed to detect whether the source of aerosol TSNAs is the liquid alone or if additional amounts may be produced due to heating. It is well known that only a portion of the TSNA in the cigarette filler is transferred to the smoke (this is also true for nicotine) and that a percentage of the TSNA found in mainstream smoke is formed during pyrolysis of the tobacco. A similar process of TSNAs formation may theoretically occur in the ECs during use, however, this is an unlikely scenario considering the far lower temperatures of evaporation compared to the temperatures involved in smoking and the absence of source for nitrite. In any case, finding similar amounts of TSNAs in liquid and aerosol verifies that the levels of exposure through aerosol inhalation are by far lower compared to smoking. Moreover, finding that TSNAs are readily delivered to the aerosol makes it important to reduce TSNAs in the EC liquids to as low as possible, by using only pharmaceutical grade nicotine. Finally, TSNAs were trapped and analyzed using a methodology validated for tobacco cigarette smoke. We did not specifically evaluate whether a fraction of TSNAs escape from the glass-fiber filter. However, considering that glass fiber filters have been shown to efficiently capture nicotine from aerosol [19] and given that nicotine and TSNAs have similar structures and boiling points, it is expected that TSNAs will also be trapped effectively by a glass fiber filter.

## 5. Conclusions

The results of this study show that exposure of EC users to TSNAs is directly associated with, and similar to, the liquid content. Evaluating liquid TSNAs levels can accurately predict the levels present in the aerosol, while use of pharmaceutical grade nicotine will probably ensure minimal exposure of consumers to TSNAs from EC use.
